# Inhibitors of Protein Convertase Subtilisin/Kexin 9 (PCSK9) and Acute Coronary Syndrome (ACS): The State-of-the-Art

**DOI:** 10.3390/jcm10071510

**Published:** 2021-04-05

**Authors:** Gabriella Iannuzzo, Marco Gentile, Alessandro Bresciani, Vania Mallardo, Anna Di Lorenzo, Pasquale Merone, Gianluigi Cuomo, Mario Pacileo, Filippo M. Sarullo, Elio Venturini, Antonello D’Andrea, Carlo Vigorito, Francesco Giallauria

**Affiliations:** 1 Department of Clinical Medicine and Surgery, “Federico II” University, 80131 Naples, Italy; margenti@unina.it (M.G.); vania.mallardo@virgilio.it (V.M.); 2 Department of Medicine and Medical Specialties, A. Cardarelli Hospital, 80131 Naples, Italy; ale.bresciani@alice.it; 3 Department of Translational Medical Sciences, “Federico II” University of Naples, Via S. Pansini 5, 80131 Naples, Italy; dilorenzoanna2@gmail.com (A.D.L.); pasqualemerone.3@gmail.com (P.M.); gianluigi.cuomo95@gmail.com (G.C.); vigorito@unina.it (C.V.); francesco.giallauria@unina.it (F.G.); 4 Unit of Cardiology and Intensive Care, “Umberto I” Hospital, Viale San Francesco, 84014 Nocera Inferiore, Italy; pacmario@yahoo.it (M.P.); antonellodandrea@libero.it (A.D.); 5 Cardiovascular Rehabilitation Unit, Buccheri La Ferla Fatebenefratelli Hospital, 90123 Palermo, Italy; sarullo.filippo@fbfpa.it; 6 Cardiac Rehabilitation Unit, Azienda USL Toscana Nord-Ovest, Cecina Civil Hospital, 57023 Cecina, Italy; vent.elio@tin.it

**Keywords:** acute coronary syndrome, PCSK9 inhibitors, cardiovascular risk

## Abstract

Acute Coronary Syndrome (ACS) remains one of the most frequent causes of morbidity and mortality in the world. Although the age- and gender-adjusted incidence of ACS is decreasing, the mortality associated with this condition remains high, especially 1-year after the acute event. Several studies demonstrated that PCSK9 inhibitors therapy determine a significant reduction of major adverse cardiovascular events (MACE) in post-ACS patients, through a process of plaque modification, by intervening in lipid metabolism and platelet aggregation and finally determining an improvement in endothelial function. In the EVACS (Evolocumab in Acute Coronary Syndrome) study, evolocumab allows >90% of patients to achieve LDL-C < 55 mg/dL according to ESC/EAS guidelines compared to 11% of patients who only receive statins. In the EVOPACS (EVOlocumab for Early Reduction of low-density lipoprotein (LDL)-cholesterol Levels in Patients With Acute Coronary Syndromes) study, evolocumab determined LDL levels reduction of 40.7% (95% CI: 45.2 to 36.2; *p* < 0.001) and allowed 95.7% of patients to achieve LDL levels <55 mg/dL. In ODYSSEY Outcome trial, alirocumab reduced the overall risk of MACE by 15% (HR = 0.85; CI: 0.78–0.93; *p* = 0.0003), with a reduced risk of all-cause mortality (HR = 0.85; CI: 0.73–0.98: nominal *p* = 0026), and fewer deaths for coronary heart disease (CHD) compared to the control group (HR = 0.92; CI: 0.76–1.11; *p* = 0.38). The present review aimed at describing the beneficial effect of PCSK9 inhibitors therapy early after ACS in reducing LDL circulating levels (LDL-C) and the risk of major adverse cardiovascular events, which was very high in the first year and persists higher later after the acute event.

## 1. Introduction

Cardiovascular diseases remain the leading cause of disease burden globally [1,2]. According to data collected by the Global Burden of Disease (GBD), in terms of frequency, mortality and costs, ACS (Acute Coronary Syndrome) remains one of the most relevant diseases worldwide; in 2019 cases of CVD increased to 523 million, CVD deaths to 18.6 million [1]. The significant impact on disability-adjusted life years (DALY), which reached 182 million DALYs in 2019 is also notable [1].

The term ACS is used to indicate many acute cardiological diseases: acute myocardial infarction, unstable angina, or even sudden death related to a fatal arrhythmia as ventricular fibrillation. Over time, the therapeutic approach to ACS has changed; an initial therapy, focused on the reduction of myocardial damage, was followed by a long-term therapy focused on the prevention of recurrent events. These are the ones to which post-ACS patients are predisposed and which are consequent to the pathophysiological mechanisms at the base of ACS, namely, plaque disruption and arterial thrombosis.

The present review aims at describing the beneficial effect of PCSK9 inhibitors therapy early after ACS in reducing LDL circulating levels (LDL-C) and the risk of major adverse cardiovascular events (MACE), which was very high in the first year (18.3%) and persists higher later after the acute event (20%) [3].

## 2. PCSK9: Direct and Indirect Role on Coronary Plaques

PCSK9 (Protein Convertase Subtilisin/Kexin 9) was first discovered in cerebellar granular neurons, and initially named Neuronal Apoptosis Regulated Convertase-1 since it is involved in the mechanism of apoptosis [4].

PCSK9 synthesis occurs mostly in the liver, but is also found in small intestine, kidney, and muscle cells.

Once produced as a 75 kDa pro-protein, the signal peptide is removed and a 3-domain heterodimer is secreted: a pro-domain, which undergoes autocatalytic cleavage; a catalytic domain, which contains a proteolytic active site; and a C-terminal domain, which consists of 3 similar modules. The catalytic domain is fundamental for the degradation function of the LDL receptor, as it interacts with the EGF-A domain of the latter [4].

Plasma PCSK9 can be predominantly found as a heterodimer (62 + 13 kDa), which is considered the active form (for its highly affinity to LDLR); it can also be found in a less active form (with 2-fold reduced affinity to LDLR), which has a lower molecular weight.

**Figure 1:**
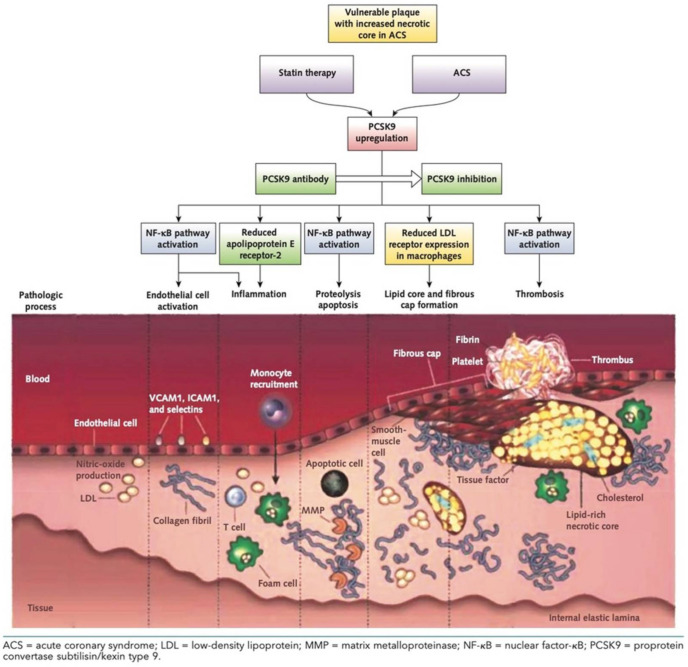
Adverse mechanisms on coronary plaque by PCSK9 (reproduced from Navarese et al., Ann Intern Med. 2016 May 3;164(9):600–7.).

Conversely, intracellular PCSK9 is only found in its proprotein form or as a ready-to-use heterodimer [5]. PCSK9 is implied both directly and indirectly in the process of atherosclerotic plaque formation. On the one hand, it acts directly through the pro-inflammatory oxidation of LDL and the modification of plaque composition for an inflammatory response mediated by cytokines, chemokines, and adhesion molecules [6] (Figure 1).

Inflammation has been shown to play a key role in the long-time atherosclerosis process leading to the formation and progression of atherosclerotic plaque [7]. In the main lesions, the finding of lipid accumulations in the middle layer of the arterial wall, following an endothelial injury, is predominant. Activated endothelial cells trigger a cytokine cascade following the expression of various adhesion molecules, including intercellular adhesion molecule-1 (ICAM-1), P-selectin, E-selectin, vascular adhesion molecule-1 (VCAM-1), attracting lymphocytes and monocytes, which infiltrate the endothelium and arterial wall (Figure 1).

Many cells type and cytokines (lymphocytes T and B, macrophages, dendritic cells, interleukins, TNF-α) are involved in this process. The whole inflammatory process leads to the oxidation of LDL molecules to oxLDL, which accumulate in the arterial wall and are phagocytosed by macrophages, which transform into foam cells, contributing to the development of atherosclerotic plaque (Figure 1).

In addition, PCSK9 can switch leukocytes into a pro-inflammatory state causing the production of pro-inflammatory cytokines, which have direct role in the development of atherosclerosis. Particularly, it was demonstrated that PCSK9 is associated with changes in T-cell programming that determines an increasing production of IL-17, which is strongly related to the development of atherosclerotic plaque [8].

Moreover, endothelial cells also express PCSK9, and moderate shear stress (3–6 dynes/cm^2^) causes production of reactive oxygen species (ROS), inducing expression of PCSK9, which itself can increase oxLDL receptors (LOX-1) expression in endothelial cells, causing an increase of cholesterol uptake in these cells [9] (Figure 1).

On the other hand, PCSK9 intervenes indirectly in atherosclerotic plaque formation by taking action in lipid metabolism and platelet aggregation. PCSK9 has direct effect on lipid metabolism by modulating the expression of the LDLR, of the very low-density lipoprotein receptor (VLDLR) and of the LDL receptor-related protein-1 (LRP1). LDL molecules are captured by the LDLR found on the hepatocyte membrane: then, through endocytosis, the LDL-LDLR complex penetrates the cell and merges with the lysosomes, where it is digested, so the LDLR can be recycled and return to the hepatocyte membrane (Figure 1).

PCSK9 increases circulating LDL levels inducing the degradation of LDLR and inhibiting recycling. PCSK9–LDLR capture, internalization, and lysosomal degradation occur within 2 to 3 h from initial contact [10]. It has been demonstrated that in hepatocytes, statins could up-regulate PCSK9 levels more than LDLR. This effect has been defined by some authors as the “statin paradox,” because statins increase LDLR activity causing lowering of the circulating LDL, but on the other side reduce the capture of circulating LDL, by increasing the expression of PCSK9 [11].

Furthermore, it has been established that PCSK9 regulates VLDLR protein levels in adipose tissue [12]. Mice with PCSK9 homozygosis gene deletion exhibit levels of VLDL until 40-fold higher, while expression of PCSK9 in same mice re-established normal levels of VLDLR, suggesting that PCSK9 could limit visceral adipogenesis and VLDLR expression in adipose tissue [12]. The result is not only the well documented reduction in LDL metabolism, increasing its plasma concentration, but also an impairment of triglycerides-rich-lipoproteins clearance [13].

Nonetheless, the role of PCSK9 as a platelet activator has only recently been demonstrated. In particular, PCSK9 directly enhances platelet activation and in vivo thrombosis by binding CD36 receptor in platelets, thus, inducing the activation of its downstream signaling pathways, independent of LDL-related pathways [14].

PCSK9, concentration-dependently, increases platelet aggregation, both in humans and mice, meanwhile this impact is significantly reduced by anti-CD36 antibody and deficiency of CD36, respectively (14). CD36, also named fatty acid translocase (FAT), is an integral membrane protein, which imports fatty acids inside the cells and binds many ligands such as collagen, thrombospondin, and oxLDL [15]. In other words, CD36 is a scavenger receptor that has already proved to be a leading actor in the progression of the atherosclerotic plaque, including foam cells formation [16]. PCSK9, through the binding to CD36 receptor, enhances several pathways downstream, which play a significant role in platelets aggregation, as well as in the genesis of the atherosclerotic plaque. In particular, CD36 recruits and activates Fyn and Lyn (members of Src family kinase), which downstream phosphorylate and activate phospholipase C gamma 2 (PLCγ2) and Syk (a non-receptor tyrosine kinase); the former leads the CD36 cascade to the activation of Protein Kinase C (PKC), a serine/threonine kinase that promotes with TRAF4 and Lyn the phosphorylation and assembly of the NOX complex, an important ROS generator [17]; ROS production activates redox-sensitive signaling pathways mediated mainly by MAPK-ERK5 (also called “Big MAP-Kinase”), and in a less significant amount by MAPK-JNK, and these redox sensors lead to procoagulant phosphatidyl-serine (PSer) externalization through a caspase cascade [18]. Moreover, CD36 recruits cytosolic phospholipase A2 (cPLA2) through the p38MAPK pathway; cPLA2 increases cytosolic concentration of arachidonic acid, and cyclooxygenase-1/thromboxane synthase (COX-1) catalyzes its conversion to thromboxane (Tx) A_2_; TxA_2_ activates glycoprotein (GP) IIb/IIIa receptors (also known as integrin alphaIIb-beta3) by synergic interactions with the cascades triggered in platelets by the binding of thrombin, adenosine diphosphate, and collagen to respective receptors; the platelets aggregation is eventually concluded through the binding of fibrinogen to activated GP IIb/IIIa receptors on contiguous platelets [19].

In the PCSK9-REACT study, including patients with ACS undergoing angioplasty and treated with ticagrelor and prasugrel, a significant association was found between PCSK9 plasma level and residual platelet activity, assessed by electrical impedance due to platelet aggregation [20]. At 1-year follow-up, 22% of patients with elevated PCSK9 plasma levels experienced a MACE, compared with 2% of patients with lower PCSK9 plasma levels (HR:2.61, 95% CI 1.24–5.52; *p* = 0.01) [20]. These findings support the idea that dosing PCSK9 levels in first phases after ACS could assist in preventing recurrence of MACE.

Furthermore, this association among plasma levels of PCSK9, platelet reactivity, and major cardiovascular events were also confirmed in a population of patients with atrial fibrillation treated with vitamin K antagonists [21].

## 3. Inhibitor of PCSK9

In recent years, the need for drugs that act on dyslipidemias, in particular on levels of circulating LDL, with a different mechanism of action from that of the widely used statins, has become increasingly evident. Statin intolerance is a very common phenomenon, and it has been documented that from 7% to 29% of patients cannot tolerate the side effects of these drugs, such as muscle pain and gastrointestinal effects [22]. Moreover, PCSK9 expression is significantly upregulated by statins [11], and this evidence suggests that the combination with PCSK9 inhibitor could potentially increase the effects of therapy. Monoclonal antibodies to PCSK9 are high molecular mass proteins (~150 kDa), which need to be injected by subcutaneous or intramuscular way. They have been shown to be effective in making the binding sites of circulating PCSK9 molecules unavailable, thus preventing the degradation of LDLR, which can thus capture circulating LDL particles and eliminate them from the bloodstream. The first anti-PCSK9 antibodies, alirocumab and evolocumab, were approved for use in USA and Europe in 2015. Therapeutic effect of PCSK9 inhibition, resulting in reduction of circulating LDL levels, in humans is evident after 2 to 3 days from start of therapy [23].

In addition to the effects on circulating LDL levels, the FOURIER (Further Cardiovascular Outcomes Research with PCSK9 Inhibition in Subjects with Elevated Risk) trial demonstrated that anti-PCSK9 could reduce Lp(a) concentration by ≈25% to 30%, and patients with higher baseline Lp(a) concentration may derive enhanced benefit from treatment [24].

Lipoprotein [Lp(a)] is a low-density lipoprotein (LDL) like particle that contains apolipoprotein(a). Lp(a) plasma concentration is mostly dependent on heritable and is controlled by the expression of the apo(a) gene. Several epidemiological studies have demonstrated that high Lp(a) plasma levels are associated with an increased coronary risk, showing a causal role of this particles in coronary atherosclerosis development [25]. Anyway, is not clear to date if reducing Lp(a) plasma levels leads to improved cardiovascular outcomes, but few therapies are available for reducing it, and this additional effect of PCSK9 inhibitors could demonstrate an increasing importance in preventing ACS. Therefore the FOURIER trial assessed a relationship between Lp(a) levels, PCSK9 inhibition with evolocumab, and CV risk reduction: achieved Lp(a) levels were significantly related to adjusted risk of CHD death, MI, or urgent coronary revascularization (HR, 1.04; 95% CI, 1.01–1.06; *p* = 0.01 per doubling of achieved Lp(a) concentration), while risk of major coronary events was reduced to a greater extent in patients with higher baseline Lp(a) levels treated with evolocumab, in particular reducing 23% in those with a baseline Lp(a) level above the median (HR, 0.77; 0.67–0.88) versus 7% for those below the median (HR, 0.93; 0.80–1.08; *p* interaction = 0.07) [24].

Regarding PCSK9 inhibition, new molecules acting directly on RNA will soon be available. Inclisiran, a small interfering RNA (siRNA), which, after being recognized by a molecular complex, inhibits the synthesis of PCSK9 inducing mRNA cleavage. In ORION-1, a phase 2 trial, a mean reduction in LDL levels between 27.9 and 41.9% was observed 180 days after a single injection of inclisiran and a mean reduction between 28.2 and 36.6% 240 days after a single injection of inclisiran (*p* < 0.001), while a mean reduction of 35.2 to 52.6% was observed at day 180 in patients who received two-doses (*p* < 0.001) [26].

In the ORION-10 trial, in patients with atherosclerotic cardiovascular disease, inclisiran showed to be able to reduce LDL levels of 52.3% (95%CI, −55.7 to 48.8; *p* < 0.001) after 510 days, while in ORION.11 trial, in patients with atherosclerotic cardiovascular disease or an atherosclerotic cardiovascular disease risk equivalent, inclisiran reduced LDL levels of 49.9% (95% CI, −53.1 to −46.6; *p* < 0.001) [27].

## 4. LDL Management in Patients after ACS

The guidelines for the management of post-ACS patients highlight, among other things, the need to reduce the LDL-cholesterol [28], which is a well-proven causal risk factor for atherosclerotic cardiovascular disease [29,30].

In particular, ACC/AHA guidelines recommend starting a more intensive lipid-lowering therapy for LDL-C ≥ 70 mg/dL (≥1.8 mml/L) [31]. Meanwhile, ESC/EAS guidelines recommend the early use of high-intensity statins and the addition of ezetimibe and anti-PCSK9 antibodies if patients with ACS do not meet both a reduction of LDL-C ≥ 50% from baseline and an LDL-C of > 1.4 mmol/L (>55 mg/dL) [28].

Several randomized controlled clinical trials and meta-analysis have shown that lipid-lowering therapies, such as statins, ezetimibe, and anti-PCSK9 antibodies [32,33,34], reduce post-ACS cardiovascular events so much better as more aggressive and earlier is the reduction of LDL-C. Especially since, with the same LDL-C reduction, the decrease in the risk of major adverse cardiovascular events (MACE) obtained with PCSK9 inhibitors is higher than the one obtained with statins [35].

The first year after an ACS is the most vulnerable period for a new event [36,37,38]. Although it is known that approximately 1 in 5 patients is at risk of having a new event during the first-year post ACS and that in elderly patients the risk is higher (approximately 2 in 5 patients), only 30% of post-ACS patients reach levels of LDL-C>mg/dL 1.8 mml/L and especially the use of PCSK9 in association with traditional therapies allows, in the latter patients, the achievement of LDL values even much lower than the target without particular safety issues.

Even though, neither evolocumab nor alirocumab have shown significant reductions in inflammatory markers in ACS patients; however, both PCSK9 inhibitors, in combination with high-intensity statins, resulted in a significant reduction in LDL-C levels after 8 weeks.

In EVACS (Evolocumab in Acute Coronary Syndrome) study, patients with ACS (non-STEMI but with troponin > 5 mg) were enrolled and treated with Evolocumab SQ 420 mg or with placebo within 24 h from the event and lipid parameters and primary endpoints were evaluated at hospital discharge and one month after it (39). Evolocumab allows >90% of patients to achieve LDL-C < 55 mg/dL according to the ESC/EAS guidelines compared to 11% of patients who only receive statins [39] (Table 1).

In EVOPACS (Evolocumab for Early Reduction of LDL Cholesterol Levels in Patients With Acute Coronary Syndromes) study, efficacy and safety of evolocumab were initiated in first phases of ACS, when patients still in hospital were evaluated [40]. EVOPACS included 308 patients hospitalized for ACS with elevated LDL levels were enrolled and randomly assigned to placebo group or receive evolocumab 420 mg on top of high intensity statin therapy, in-hospital and after 4 weeks. Evolocumab showed to reduce LDL levels of 40.7% (95% CI: 45.2 to 36.2; *p* < 0.001) and allows 95.7% of patients to achieve LDL levels < 55 mg/dL (40) (Table 1).

In the ODYSSEY Outcome study, they randomized patients from 1 to 12 months out from an ACS event (undergoing percutaneous or surgical myocardial revascularization) with LDL-C values of at least 70 mg/dL, already being treated with high-intensity statins or at maximum tolerated doses, treated with Alirocumab 75/150 mg every 2 weeks or with placebo for 64 weeks and evaluated for lipid parameters and endpoint for almost 3 years (median of 2.8 years) [41]. The primary endpoint, which included heart attack, ischemic stroke, death from coronary artery disease, and hospitalization for unstable angina, was reached because Alirocumab reduced the overall risk of MACE by 15% (HR = 0.85; CI: 0.78–0.93; *p* = 00003), with a reduced risk of all-cause mortality (HR = 0.85; CI: 0.73–0.98: nominal *p* = 0026), and fewer deaths for CHD compared to the control group (HR = 0.92; CI: 0.76–1.11; *p* = 0.38) [41] (Table 1).

Moreover, it should not be overlooked that despite being targeted according to the guidelines, new events occur in a not-small percentage of post-ACS patients. The clinical picture of “residual risk” is outlined, to which small and dense LDL [42] and Lp(a) [43], that are now well-known residual risk factors, contribute in a decisive way and to the reduction of which also anti-PCSK9 antibodies contribute, even with a mechanism which is still not clear.

## 5. Pitfalls and Future Perspectives

Although most of patients receiving PCSK9 inhibitors reach the expected 50–60% reduction in LDL-C, sporadically a limited LDL-C lowering response is reported [44]. Several mechanisms have been proposed for PCSK9 inhibitors hypo-responsiveness (<15% of LDL-C reduction): (1) Impaired monoclonal antibody entry into the systemic circulation; and (2) physiological impairment once the monoclonal antibody is absorbed. Poor adherence to therapeutic protocols; improper administration; dermatological factors impairing systemic absorption of drug; and inappropriate antibody disposition may be at the basis of the reduced entry of PCSK9 inhibitors into the systemic circulation [45,46].

Conversely, mutations altering the antibody binding site on circulating PCSK9; anti-drug antibodies directed against PCSK9 inhibitors; exaggerated PCSK9 secretion; and mutations and/or dysfunctional LDLR, apoB, and/or apoE might affect circulating therapeutic levels of PCSK9 inhibitors. However, it should be noted that the most common cause of apparent PCSK9 inhibitors resistance is related to the discontinuation of concurrent lipid lowering therapies after initiation of PCSK9 inhibitors. Other novel drugs are currently at various stages of development. Preliminary reports demonstrate similar effect on lipoprotein parameters but highlight other potential advantages over PCSK9i monoclonal antibodies. Inclisiran, a small interfering ribonucleic acid (siRNA) oligonucleotide, inhibits the translation of PCSK9 mRNA into biologically active protein [47]. The main effect consists of inhibition of intracellular production of PCSK9, causing lower plasma PCSK9 and without the compensatory increases in total plasma PCSK9 seen with monoclonal antibody interventions. This peculiar aspect still remains to be elucidated [45]. Notably, the siRNA technology allows for prolongation of dosing intervals (at day 0, 90, then every 180 days thereafter), an attractive pharmacokinetic property for medication adherence improvement. Oral small-molecule inhibitors, vaccination, and clustered regularly interspaced short palindromic repeats (CRISPR) are novel strategies for PCSK9 lowering. The latter two interventions, pave the way for primordial care, counteracting the development of the risk factor before it manifests [48].

## 6. Conclusions

PCSK9 inhibitors therapy shows a significant reduction of MACE in post-ACS patients. This seems to be achieved both directly through a process of modification in plaque composition with its subsequent stabilization and indirectly by intervening in lipid metabolism and platelet aggregation. In addition, PCSK9 inhibitors determine an improvement in endothelial function in patients with familial hypercholesterolemia, an iconic population for premature cardiovascular disease [49,50].

Early introduction of PCSK9 inhibitors for a more rapid and aggressive approach for MACE prevention is strongly recommended since cardiovascular risk remains high during the first year after ACS and increases overtime.

## Figures and Tables

**Table 1:** Key results of low-density lipoprotein (LDL)-cholesterol management with PCSK9 inhibitors after acute coronary syndrome.

Study ID	Study Population/Design	Changes in LDL (%)	Efficacy	Safety
EVACS [39]	56 patients: 29 evolocumab, 27 placebo; Mean age 55 ± 13; Male 59%;	−61% after 30 days.	LDL-C in patients treated with evolocumab was 28.6 mg/dL lower than in the placebo group after 30 days; >75% patients have LDL-C < 55 mg/dL after 30 days.	
EVOPACS [40]	308 patients: 155 evolocumab, 153 placebo; Mean age 60.5 ± 12 (evolocumab), 61.0 ± 10.7 (placebo); Male 83% (evolocumab), 80% (placebo)	−77.1 ± 15.8% after 8 weeks	LDL mean difference reduction of −40.7% between evolocumab and placebo group (95% CI: −45.2 to −36.2%; *p* < 0.001). Lp(a) mean difference reduction of −10.4% (*p* = 0.47)	Similar between two groups. Local injection site reactions: 3.2% (evolocumab), 2% (placebo); All-cause death: 1.3% (evolocumab), 0% (placebo) (*p* = 0.5)
ODYSSEY [41]	18924 patients: 9462 alirocumab, 9462 placebo; Mean age: 58.5 ± 9.3 (alirocumab), 58.6 ± 9.4 (placebo); Male: 74.7% (alirocumab), 74.9% (placebo);	−62.7% after 4 months,−54.7% at 48 months after randomization	Reduction of 15% of MACE (HR: 0.85, 95%CI: 0.78–0.93, *p* = 0.0003, absolute risk reduction: 1.7% CHD death was not significantly reduced (HR: 0.92, 95%CI: 0.76–1.11, *p* = 0.38) Non-fatal MI: HR: 0.86 (95%CI: 0.77–0.96, *p* = 0.006) Ischemic stroke: HR: 0.73 (95%CI: 0.57–0.93, *p* = 0.01) Unstable angina: HR: 0.61 (95%CI: 0.41–0.92, *p* = 0.02)	Similar between two groups. Local injection site reactions: 3.8% (alirocumab), 2.1% (placebo). ALT > 3 times U.L.N.: 2.3 (alirocumab), 2.4 (placebo); Adverse event that led to death: 1.9% (alirocumab), 2.4% (placebo).
